# A Downmodulated MicroRNA Profiling in Patients with Gastric Cancer

**DOI:** 10.1155/2017/1526981

**Published:** 2017-05-04

**Authors:** Tao Zhang, Chang Liu, Shi Huang, Yuanping Ma, Jiansong Fang, Yuanneng Chen

**Affiliations:** Department of Gastroenterology, Ruikang Hospital, Guangxi Traditional Chinese Medical University, Nanning, Guangxi 530011, China

## Abstract

*Objective*. Here, we aim to investigate the microRNA (miR) profiling in human gastric cancer (GC). *Methods*. Tumoral and matched peritumoral gastric specimens were collected from 12 GC patients who underwent routine surgery. A high-throughput miR sequencing method was applied to detect the aberrantly expressed miRs in a subset of 6 paired samples. The stem-loop quantitative real-time polymerase chain reaction (qRT-PCR) assay was subsequently performed to confirm the sequencing results in the remaining 6 paired samples. The profiling results were also validated in vitro in three human GC cell lines (BGC-823, MGC-803, and GTL-16) and a normal gastric epithelial cell line (GES-1). *Results*. The miR sequencing approach detected 5 differentially expressed miRs, hsa-miR-132-3p, hsa-miR-155-5p, hsa-miR-19b-3p, hsa-miR-204-5p, and hsa-miR-30a-3p, which were significantly downmodulated between the tumoral and peritumoral GC tissues. Most of the results were further confirmed by qRT-PCR, while no change was observed for hsa-miR-30a-3p. The in vitro finding also agreed with the results of both miR sequencing and qRT-PCR for hsa-miR-204-5p, hsa-miR-155-5p, and hsa-miR-132-3p. *Conclusion*. Together, our findings may serve to identify new molecular alterations as well as to enrich the miR profiling in human GC.

## 1. Introduction

Gastric cancer (GC) is among the most common cancers and second leading cause of global cancer mortality [1, 2]. Surgical resection still remains the most promising intervention towards localized GC tumors, whereas the advanced-stage GC patients who develop recurrent diseases exhibit extremely poor quality of life as well as survival rates. Scientists have identified various genetic alterations that affect GC tumorigenesis and progression over the past decade [3, 4]. To date, however, genetic markers for GC tumorigenesis or progression have not been identified.

MicroRNAs (miRs), a class of small noncoding RNAs, play an important role in a variety of cellular processes, including cell development, differentiation, apoptosis, and proliferation [5–7]. miRs exert negative genetic regulation by binding to complementary sites in the 3′-untranslated regions of the targeted messenger RNAs (mRNAs) [8]. Recently, miRs have been implicated in cancer development by acting as either tumor oncogenes or suppressors [9–11]. Accumulating body of evidence shows that miRs are significantly modulated in nearly all types of tumors, including human GC [12]. Moreover, the altered levels of miRs in tumors highly correlate with progression as well as prognosis of human tumors [13–15]. Interestingly, reconstitution of tumor suppressor miRs [16], or sequence-specific knockdown of oncogenic miRs [17], has produced favorable antitumor outcomes. Therefore, miRs are possible therapeutic targets for human tumors, including GC.

In the present study, we investigated the miR profiling in a small group of Chinese GC patients by applying a high-throughput miR sequencing method and then validated the results by performing stem-loop qRT-PCR in vivo and in vitro.

## 2. Materials and Methods

### 2.1. Ethics Statement

The study was approved by the Ethics Committee of Ruikang Hospital of Guangxi Traditional Chinese Medical University (Guangxi, China). All participants were explained their participation rights and signed the written informed consent.

### 2.2. Study Population

We recruited 12 GC patients from the Affiliated Ruikang Hospital of Guangxi University of Chinese Medicine between 2014 and 2015. They were free of any other cancers. After routine surgery, we collected tissue samples from the tumor lesion as well as from the peritumoral mucosa, which was defined as tissue that is located at 2 cm from the resection margin of the tumor lesion, from each participant. The TNM staging system confirmed that all the cancer samples were at T1N0M0 stage. And we reported here that all the cancer samples were collected from the gastric antrum, with none free of *Helicobacter pylori*. H&E stain indicated a 100% of gastric adenocarcinoma for the samples. In order to identify the differentially expressed miRs between the tumoral and the peritumoral samples, we first constructed miR sequencing library on 6 participants (3 females and 3 males, aged 52 to 71 years old) with gastric tumors and matched peritumoral tissues and then screened the differentially expressed miRs between them. Then, we validated the results on the remaining 6 patients (3 females and 3 males, aged 56 to 70 years old) using the stem-loop quantitative real-time polymerase chain reaction (qRT-PCR) assay.

### 2.3. Cell Cultures

Three human GC cell lines (BGC-823, MGC-803, and GTL-16) as well as a normal gastric epithelial cell line (GES-1) were cultured in DMEM medium (Invitrogen, Carlsbad, CA, USA) supplemented with 10% fetal bovine serum, streptomycin (100 *μ*g/ml), and penicillin (100 U/ml) at 37°C in an incubator with 5% CO_2_.

### 2.4. RNA Isolation and Quality Control

Total RNA was isolated using the mirVana™ miRNA Isolation Kit (Ambion, Austin, TX, USA) according to the manufacturer's protocol. Notably, the cells indicated above were washed for 3 times with phosphate-buffered saline before RNA extraction. The concentration and purity of RNA samples were determined by a NanoDrop ND-1000 spectrophotometer (Nanodrop Technologies, Wilmington, DE, USA).

### 2.5. High-Throughput miR Sequencing Method

We performed the high-throughput miR sequencing on 6 patients with gastric tumors and matched peritumoral tissues. The miR sequencing library was constructed by applying the Illumina TruSeq Small RNA Sample Prep Kit Set A (San Diego, CA, USA). Briefly, total RNA of each sample was used to prepare the miR sequencing library, which included the following steps: (1) 3′-adaptor ligation, (2) 5′-adaptor ligation, (3) cDNA synthesis, (4) PCR amplification, and (5) size selection of ~135–155 bp PCR-amplified fragments (corresponding to ~15–35 nt small RNAs). The libraries were denatured as single-stranded DNA molecules, captured on Illumina flow cells, amplified in situ as clusters, and finally sequenced for 36 cycles on the Illumina HiSeq™ 2000 System (San Diego, CA, USA) as per the manufacturer's instructions. After sequencing, the Solexa CHASTITY quality-filtered reads were harvested as clean reads. The adaptor sequences were trimmed and the adaptor-trimmed reads (≥15 nt) were left. miRDeep2 software was used to predict the novel miRs with these trimmed reads [18]. Then, the trimmed reads were aligned to merged pre-miRNA databases (known pre-miR from miRBase v21 plus the newly predicted pre-miRs) using Novoalign software v2.07.11 (http://www.novocraft.com) with at most one mismatch. The numbers of mapped tags were defined as the raw expression levels of that miR. To correct for the difference in tag counts between samples, the tag counts were scaled to TPM (the copy number of transcripts per million) based on the total number of tag aligned [19]. Fold change and *p* value were calculated and used to identify the differentially expressed miRs between tumoral and peritumoral tissues. The miRs, which matched *p* < 0.05 and fold change > 2 (upregulated) or <0.5 (downregulated), were considered differentially expressed miRs.

### 2.6. qRT-PCR Validation

In order to further validate the results of the differentially expressed miRs identified by the high-throughput miR sequencing method between the tumoral and peritumoral tissues, the stem-loop qRT-PCR method using a mirVana miRNA Detection Kit and gene-specific primers (Ambion) was introduced to quantify the expression levels of the miRs in vivo on the remaining 6 GC patients as well as in vitro on the cell lines indicated above [20]. The PCR reactions (20 *μ*l) were performed in 96-well plates and run in triplicate on a Bio-Rad CFX96 Touch Real-Time PCR Machine. Thermal cycling was organized in 3 repeated steps: the first denaturation step of 95°C for 10 minutes, followed by 40 cycles of 95°C for 15 seconds, and 60°C for 1 minute. RNU6B was used as endogenous controls to normalize the relative expression levels of the miRs.

### 2.7. Statistical Analyses

STATISTICA 10 (Dell, Round Rock, TX, USA) was used to perform the statistical analyses. Graph preparations were carried out by Microsoft Office Excel 2007. Student *t*-tests were applied to compare data between groups. A *p* value less than 0.05 was considered to be statistically significant.

## 3. Results

### 3.1. RNA Quality Control

Because construction of miR sequencing library requires high-quality RNA samples, thus we would not consider those that failed to meet OD_260/280_  ratio > 1.8 as well as OD_260/230_  ratio > 1.8. We show in Table 1 that all the RNA samples used in the present study were tested qualified for the construction of miR sequencing library.

### 3.2. Quality Assessment of the miR Sequencing Library

Quality assessment of the sequencing library was determined by an Agilent 2100 Bioanalyzer using the Agilent DNA 1000 chip kit (Santa Clara, CA, USA). We selected size of ~135–155 bp PCR-amplified fragments. The library, of which the concentration was below 1.0 ng/*μ*l, would not be taken into consideration.  Table 2 displays that all the libraries were tested qualified.

### 3.3. Altered miR Expression Pattern in Human GC

**Figure 1:**
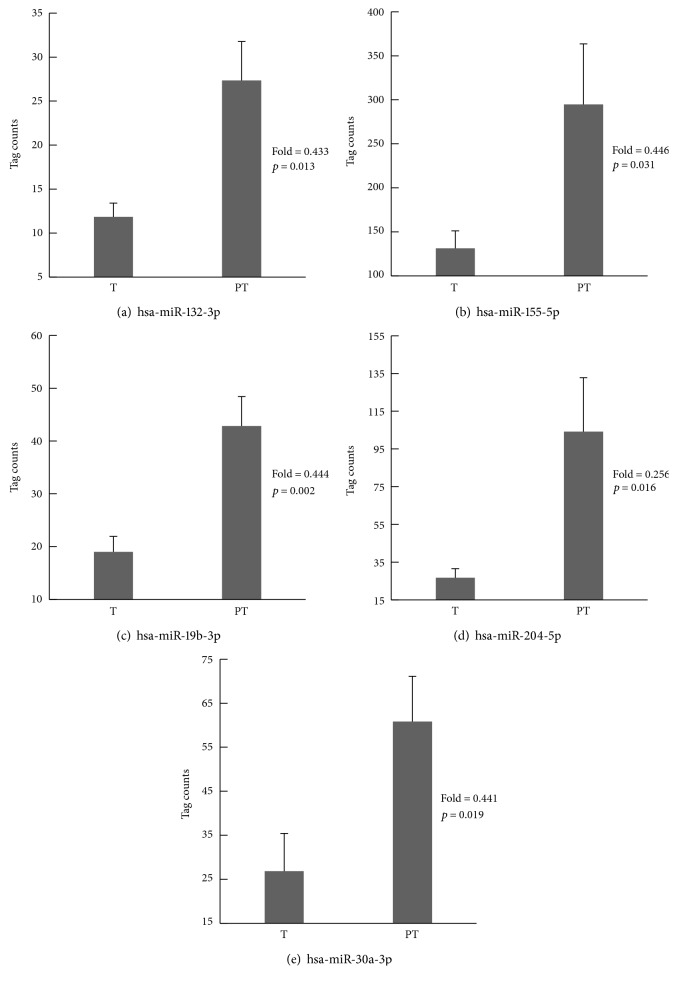
Five downregulated miRs between gastric tumor (T) and peritumoral tissue (PT) were identified by the high-throughput miR sequencing method. Tag counts refers to the normalized tag number of the mature miRs annotated in miRBase 21 (TPM), including the tag number of all samples. A fold change less than 0.5 (as well as *p* value less than 0.05) indicated that the expression of the miR was downregulated in gastric tumor compared with the corresponding adjacent tissue. Data are represented as mean ± SD, *n* = 6.

First, we attempted to screen the expression of human miRs of 6 GC specimens and of their matched peritumoral tissues using the high-throughput miR sequencing method. This approach allowed us to identify 5 differentially expressed miRs, hsa-miR-132-3p (A, *p* = 0.013), hsa-miR-155-5p (B, *p* = 0.031), hsa-miR-19b-3p (C, *p* = 0.002), hsa-miR-204-5p (D, *p* = 0.016), and hsa-miR-30a-3p (E, *p* = 0.019), that were significantly modulated between tumoral and peritumoral tissues (Figure 1). Furthermore, the altered miR expression showed a downregulated pattern in human GC, as confirmed by the fold changes. Interestingly, the altered miR expression pattern was consistent with the previous studies for hsa-miR-155-5p [21, 22], hsa-miR-19b-3p [23], and hsa-miR-204-5p [15, 24] in human GC.

**Figure 2:**
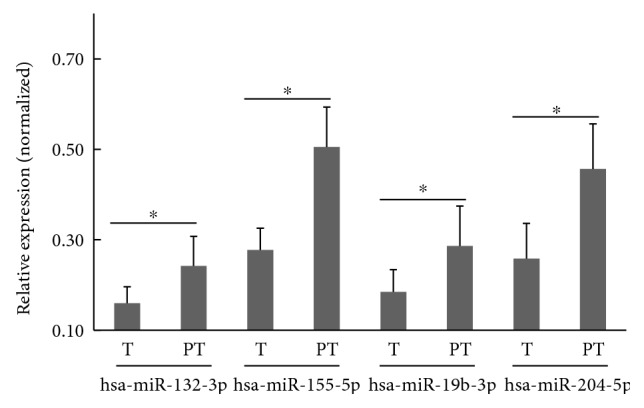
Stem-loop qRT-PCR confirmed the altered expression pattern of four miRs between tumoral (T) and peritumoral tissues (PT) in vivo. The relative miR expression levels were normalized by RNU6B. Data are expressed as mean ± SD, *n* = 6. An asterisk (^∗^) indicates *p* < 0.05.

In order to further confirm the miR sequencing results, we then performed stem-loop qRT-PCR on the remaining 6 GC patients for the 5 identified miRs between tumoral and peritumoral tissues. Consistent with the miR sequencing results, we observed that hsa-miR-132-3p (*t* = −2.690, *p* = 0.023), hsa-miR-155-5p (*t* = −5.553, *p* = 0.000), hsa-miR-19b-3p (*t* = −2.458, *p* = 0.034), and hsa-miR-204-5p (*t* = −3.843, *p* = 0.003) were significantly downmodulated in human GC compared with peritumoral tissues (Figure 2). However, the expression of hsa-miR-30a-3p remained unaltered (data not shown).

**Figure 3:**
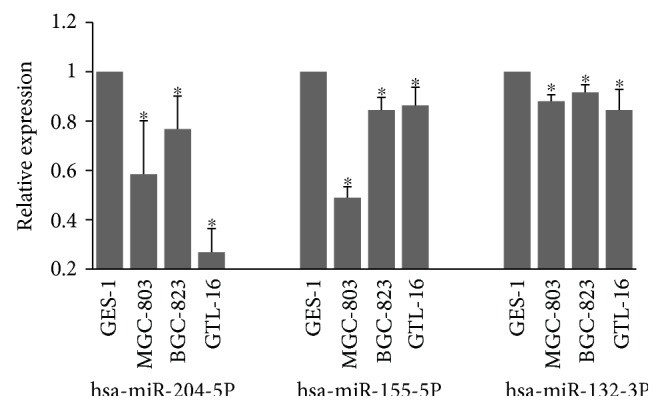
The expression levels of hsa-miR-204-5p, hsa-miR-155-5p, and hsa-miR-132-3p were relatively downregulated in three human GC cell lines. The expression levels of the three GC cell lines (MGC-803, BGC-823, and GTL-16) were calculated as relative expression to that of the control group, that is, the normal gastric epithelial cell line (GES-1). Experiments were performed in triplicate. Data are expressed as mean ± SD. An asterisk (^∗^) indicates *p* < 0.05 (versus that in GES-1).

As shown above, the miR sequencing and qRT-PCR results agreed with each other for the modulation pattern of hsa-miR-132-3p, hsa-miR-155-5p, hsa-miR-19b-3p, and hsa-miR-204-5p in human GC. We speculated that similar result(s) might probably be obtained in vitro as well. To achieve this, we next investigated the expression levels of the 5 indicated miRs in three human GC cell lines (BGC-823, MGC-803, and GTL-16) and a normal gastric epithelial cell line (GES-1). Interestingly, we observed a strong difference of the expression levels of hsa-miR-204-5p in all three human GC cell lines, MGC-803 (*t* = 4.261, *p* = 0.002), BGC-823 (*t* = 4.694, *p* = 0.001), and GTL-16 (*t* = 18.544, *p* = 0.000), when compared with that in the normal gastric epithelial cell line, GES-1 (Figure 3). Similar results were also observed for hsa-miR-155-5p (MGC-803: *t* = 20.281, *p* = 0.000; BGC-823: *t* = 5.286, *p* = 0.006; and GTL-16: *t* = 3.196, *p* = 0.033) as well as for has-miR-132-3p (MGC-803: *t* = 7.755, *p* = 0.001; BGC-823: *t* = 4.707, *p* = 0.009; and GTL-16: *t* = 3.314, *p* = 0.032), as shown in Figure 3. However, we failed to replicate the in vivo findings of hsa-miR-19b-3p or has-miR-30a-3p in the present three GC cell lines (data not shown). Taken together, our data demonstrated a downregulated miR profiling in human GC tissue, which was further agreed by in vitro results for hsa-miR-204-5p, hsa-miR-155-5p, and hsa-miR-132-3p.

## 4. Discussion

miRs may act as either tumor suppressors or oncogenes in cancer development [9–11]. Accumulating body of evidence highlights miRs as molecular targets whose modulation may hold therapeutic promise towards different types of tumor [25], including GC [26]. Hundreds of differentially expressed miRs have been reported between GC specimens and their matched peritumoral tissues over the past decade [12]. Downregulated miRs frequently lead to loss of tumor suppressor activity. A recent study indicated that downregulation of a large amount of miRs played a critical role in tumorigenesis independently from the type of tumor analyzed [27]. In the present study, we identified 4 significantly downregulated miRs, hsa-miR-204-5p, hsa-miR-155-5p, hsa-miR-132-3p, and hsa-miR-19b-3p, in human GC tissues by a combination of the high-throughput miR sequencing approach as well as subsequent qRT-PCR validation. We also observed a downmodulated level of hsa-miR-204-5p, hsa-miR-155-5p, and hsa-miR-132-3p in three human GC cell lines (BGC-823, MGC-803, and GTL-16) when compared with that in a normal gastric epithelial cell line (GES-1).

Accumulating studies demonstrated that the expression of miR-204 was drastically downmodulated in various types of cancer, including ovarian cancers, colorectal cancer, pediatric renal tumors, breast cancers, and glioblastoma [28–30]. In the current study, we showed a similar result in human GC as well as in several GC cell lines, which was further agreed by several other in vivo and in vitro studies [15, 24, 31]. For example, Zhang et al. demonstrated that miR-204-5p was significantly downmodulated in a GC cohort [24]. A very recent review believes that miR-204 acts as a tumor suppressor via promoting apoptosis, decreasing resistance of cancer cells to chemotherapy, and suppressing the self-renewal of cancer stem cells [32]. Interestingly, several research groups tentatively documented the underlying mechanisms that how miR-204 functions in GC development [15, 24, 31, 33]. For example, Sacconi et al. demonstrated that miR-204 targeted B cell lymphoma-2 (Bcl-2) mRNA and increased responsiveness of GC cells to 5-fluorouracil and oxaliplatin treatment, and ectopic expression of Bcl-2 counteracted miR-204 proapoptotic activity [15]. Another study evidenced that a downmodulation of miR-204 promoted GC cell invasion by activating the SIRT1- (sirtuin type 1)-LKB1 (liver kinase B1) pathway [31]. Zhang et al. identified that ubiquitin-specific peptidase 47 (USP47) and Ras-related protein Rab-22A (RAB22A) were direct functional targets of miR-204-5p in GC and that miR-204-5p inhibited GC cell proliferation by downregulating USP47 and RAB22A [24]. Together, these results indicate that regulation of miR-204 expression may hold therapeutic promise for human GC.

miR-155 is primarily expressed within lymphocytes and functions as an important regulator of the immune system [34]. The underlying mechanisms have been well documented elsewhere [35, 36]. Recent studies have shown that transgenic mice expressing miR-155 in B lymphocytes generated lymphoma, indicating that miR-155 is oncogenic [37, 38]. This is further agreed by an accumulating body of clinical evidence showing that several types of malignancy express high level of miR-155. For instance, overexpression of miR-155 is detected in a number of B cell lymphomas [39–41]. Moreover, the oncogenic role of miR-155 is also described in several other cancers where overexpression of miR-155 frequently corresponds with poor prognosis [42–44]. However, we noticed that several miR profiling studies consistently reported a downmodulated alteration of miR-155 in GC tissues [45–47]. In the current study, we also observed similar result that miR-155 was aberrantly downmodulated in GC samples. The inconsistency of miR-155 expression pattern might be explained by the differences of tumor types and/or immune functions. More studies need to be taken to further address the biological functions of the miR in tumorigenesis.

Recent in vivo and in vitro investigations evidenced that miR-132 is involved in the development of several types of cancers, including breast cancer [48], ovarian cancer [49], lung cancer [50], and colorectal cancer [51]. Consistently, these studies agreed that the alteration of miR-132 showed a downregulated pattern. Interestingly, we also detected a similar result in GC tissues in the present study. Together with our study, these results indicate that miR-132 might act as a tumor suppressor. Moreover, several research groups tentatively explored the underlying mechanisms of miR-132 in cancer development. For example, Tian et al. revealed in vitro that miR-132 inhibited cell proliferation, invasion, and migration in ovarian cancer by targeting the transcription factor E2F5 [49]. You et al. showed that miR-132 suppressed the migration and invasion of human non-small-cell lung cancer cells via targeting the EMT- (epithelial-to-mesenchymal transition-) related transcription factor ZEB2 [50]. Similar mechanism was also reported elsewhere that miR-132 inhibited colorectal cancer cell invasion and metastasis via directly targeting ZEB2 [51]. However, little is known about the underlying mechanisms that how miR-132 contributes to GC development, which calls for future investigations.

miR-19b is fully evidenced in literature as a tumor oncogene in various types of cancers, and the underlying mechanisms are investigated accordingly [52–55]. It is also considered a novel potential biomarker to indicate progression of GC [23]. However, we failed to observe a similar result in GC tissue with a small Chinese population or in vitro with several GC cell lines in the present study. Instead, our data showed a downmodulated alteration in vivo, which indicates that miR-19b might act as a tumor suppressor towards GC. We, for the present, are unable to explain the inconsistency. A further study needs to be taken to revalidate the miR-19b expression pattern in GC.

The study has an obvious limitation, which is attributed to the small sample size. Besides, among different miR profiling studies on GC (as well as on other cancers), a certain variability of the profiling results exists and these discrepancies could be probably related to some factors, for example, test sample size and biological and analytical differences. Thus, findings and/or conclusions of the present study should be considered preliminary until replicated studies with larger sample sizes are conducted in the future.

Taken together, we in vivo and in vitro detected a set of downmodulated miRs in human GC, most of which were consistent with a plenty of previous studies investigating other types of cancer. Our results may contribute to the identification of new molecular alterations as well as to the enrichment of the miR profiling in human GC.

## Figures and Tables

**Table 1:** Quality control of RNA samples for the construction of miR sequencing library.

Sample ID	OD_260/280_ ratio	OD_260/230_ ratio	Conc. (ng/*μ*l)
T-1	1.95	2.25	1218
T-2	1.92	2.27	960
T-3	1.96	2.28	1850
T-4	1.99	2.32	1348
T-5	1.99	2.30	1671
T-6	1.97	2.30	1273
PT-1	1.88	2.11	1327
PT-2	1.92	2.18	1590
PT-3	1.94	2.26	1789
PT-4	1.94	2.29	1430
PT-5	1.81	1.81	1350
PT-6	1.81	1.81	1106

T: gastric tumor; PT: peritumoral tissue.

**Table 2:** Quality assessment of the miR sequencing library.

Sample ID	Size (bp)	Conc. (ng/*μ*l)	Volume (*μ*l)	Total amount (ng)
T-1	147	1.94	20	38.8
T-2	149	1.66	20	33.2
T-3	147	2.09	20	41.8
T-4	146	1.11	20	22.2
T-5	139	1.87	20	37.4
T-6	147	1.29	20	25.8
PT-1	148	1.63	20	32.6
PT-2	147	1.43	20	28.6
PT-3	146	1.33	20	26.6
PT-4	145	1.62	20	32.4
PT-5	146	1.90	20	38.0
PT-6	145	1.04	20	20.8

T: gastric tumor; PT: peritumoral tissue.
